# Analysis of polypharmacy effects in older patients using Japanese Adverse Drug Event Report database

**DOI:** 10.1371/journal.pone.0190102

**Published:** 2017-12-21

**Authors:** Junko Abe, Ryogo Umetsu, Hiroaki Uranishi, Honami Suzuki, Yuri Nishibata, Yamato Kato, Natsumi Ueda, Sayaka Sasaoka, Haruna Hatahira, Yumi Motooka, Mayuko Masuta, Mitsuhiro Nakamura

**Affiliations:** 1 Laboratory of Drug Informatics, Gifu Pharmaceutical University, Daigaku-nishi, Gifu, Japan; 2 Medical Database Co., Ltd., Higashi, Shibuya-ku, Tokyo, Japan; Kyushu University, JAPAN

## Abstract

Population aging is a global phenomenon, and choosing appropriate medical care for the elderly is critical. Polypharmacy is suspected to increase the risk of adverse events (AEs) in older patients. We examined the AE profiles associated with polypharmacy and aging using the Japanese Adverse Drug Event Report (JADER) database. We attempted to mitigate the effect of patient-related factors using a multiple-logistic regression technique and data subsetting. We selected case reports for AEs as specified in the Medical Dictionary for Regulatory Activities (MedDRA). The association between polypharmacy and “renal disorder” or “hepatic disorder” was evaluated using reporting odds ratio (ROR) and adjusted for covariates using multiple-logistic regression. For renal disorder, advanced polypharmacy showed higher adjusted RORs, because the value of administered drugs group [1.82 (1.76–1.88), ≥ 10] was higher than that of the number of administered drugs group [1.27 (1.24–1.31), 5–9]. The lower limit of the 95% confidence interval (CI) of adjusted ROR for age (≥ 60 years) was > 1 for renal disorder. For hepatic disorder, the adjusted RORs were as follows: 1.17 (1.14–1.20) for the number of administered drugs group (5–9) and 1.14 (1.11–1.18) for the number of administered drugs group (≥ 10). The adjusted RORs of hepatic disorder compared to those of renal disorder had lower adjusted RORs related to the increase in the number of administered drugs. Therefore, elderly individuals should be closely monitored for the occurrence of renal disorder when they are subjected to polypharmacy. This approach might apply to the simultaneous evaluation of the AE risk of polypharmacy and aging.

## Introduction

Aging of the population is a global phenomenon, and appropriate medical care for the elderly should be ensured [1–6]. A survey by the United Nations (UN), revealed that there is one older adult (≥ 60 years) in every nine people [7,8]. This value is expected to increase to one in five people by 2050 [7,8]. In the US, the > 65-year-old population, which is the fastest growing age group, has increased by 12.5% since 1999, reached approximately 40 million in 2009, and there will be approximately 72 million older adults by 2030 [2,3]. In Japan, 27.3% of the total population was aged ≥ 65 years in 2016, and this trend is expected to continue for the next 25 years [5,6].

The elderly often have multiple diseases and, therefore, are administered numerous medications, which is referred to as polypharmacy [9–11]. In the US, the proportion of people aged ≥ 65 years who were administered five or more medications tripled from 12.8% to 39.0% between 1988 and 2010 [12]. In the UK, the proportion of people aged ≥ 65 years who were administered 10 or more drugs more than tripled between 1995 (4.9%) and 2010 (17.2%) [13]. The Slone Survey reported that 60% and approximately 20% of elderly patients in the US receive more than five and 10 or more medicines, respectively [14,15]. Consequently, polypharmacy is common among the elderly [9–11].

A standard definition has not been determined for polypharmacy, which could be appropriate or problematic. When medicine use is optimized, prescriptions are based on the best evidence, which improves the patient’s life expectancy and quality of life (QOL), and is considered appropriate polypharmacy. However, when the risk of interactions and adverse events (AEs) of multiple drug cotreatments affects patients’ QOL and compliance, this is considered problematic polypharmacy [16]. Despite its benefits, polypharmacy is generally considered to increase the risk of AEs, drug-drug interactions, inappropriate prescribing, nonadherence to drug regimens, hospitalization, and mortality in the elderly [2,11]. The coadministration of at least two to nine medications is considered polypharmacy [2,17–21]. Generally, the administration of ≥ 5 or ≥ 10 prescription drugs is defined as polypharmacy or “major” polypharmacy, respectively, and based on these thresholds, serious AEs and drug interaction have been observed in both outpatients and inpatients [13,22–25].

Increased risk of AEs in the elderly might cause physiological changes related to aging that alter pharmacokinetic and pharmacodynamic drug responses [26]. In particular, alternations in hepatic and renal function are considered to be responsible for the change in pharmacokinetics in the elderly [27]. Aging decreases hepatic metabolism because of reduced liver mass, hepatic blood flow, and albumin synthesis [27]. Aging also decreases renal drug elimination because of reduced glomerular filtration rate (GFR) and tubular function [27]. We considered that the evaluation of AEs related to polypharmacy and associated with hepatic and renal disorders would be an extremely interesting subject.

The spontaneous reporting system (SRS) of the real-world setting has been used in pharmacovigilance assessments [28–31]. The US Food and Drug Administration (FDA) adverse event reporting system (FAERS) is an SRS and the largest database in the world [28–31]. Based on the FAERS database, we previously reported that polypharmacy with antipsychotic drugs might increase the risk of hyperglycemic AE using well-established pharmacovigilance indexes such as the reporting odds ratio (ROR) [32]. The regulatory authority in Japan, the Pharmaceuticals and Medical Devices Agency (PMDA), controls the SRS of the Japanese Adverse Drug Event Report (JADER) database which is a best-known SRS in Japan. The JADER database for individual case safety reports is similar to the FAERS database and is used to assess the risk of AEs. However, investigations of possible associations between polypharmacy and AE are rare using the JADER database.

The effect of aging and polypharmacy, such as the number of administered drugs, on AEs was evaluated separately in the previous studies [2,17,24,33]. In this present study, we evaluated the concomitant effects of aging and polypharmacy on AEs of hepatic and renal disorders using adjusted RORs and a multiple-logistic regression technique in the JADER database.

## Materials and methods

### Data sources

Data from the JADER database, which contains data recorded from April 2004 to June 2017, were obtained from the PMDA website (www.pmda.go.jp). All data of the JADER database were fully anonymized by the regulatory authority before we accessed them. The structure of the JADER database complies with the international safety reporting guidelines (International Concil on Harmonisation [ICH] E2B). The database consists of four data tables: 1) patient demographic information (DEMO), 2) drug information (DRUG), 3) AEs (REAC), and 4) primary illness (HIST). The JADER database does not contain the codes for identifying case reports (A1.11) and, therefore, we could not exclude duplicate cases from the same patient (https://www.pmda.go.jp/files/000145474.pdf). In the DRUG table, the causality of each drug was assigned a code according to its association with the AEs such as a “suspected drug,” “concomitant drug,” or “interacting drug.” All drugs in the “suspected drug,” “concomitant drug,” and “interacting drug” association classes were used for the analyses.

The description of age is recorded in the data table of DEMO that includes patient demographic information. The specific items entered in the DEMO data table are as follows: < 10, 10–19, 20–29, 30–39, 40–49, 50–59, 60–69, 70–79, 80–89, 90–99, ≥ 100 years, neonate, baby, infant, child, young adult, adult, elderly, first trimester, second trimester, third trimester, and unknown. The reports were stratified by age as follows: ≤ 19, 20–29, 30–39, 40–49, 50–59, 60–69, 70–79, 80–89, and ≥ 90 years. The stratified ≤ 19-year-old age group for the analysis combined the < 10, 10–19 years, neonate, baby, infant, and child groups. The stratified ≥ 90-year-old group consisted of the 90–99 and ≥ 100-year-old. We excluded the following items, young adult, adult, elderly, first trimester, second trimester, third trimester, and unknown because these descriptions could not be categorized into precise 10-year intervals.

Most developed world countries and the World Health Organization (WHO) have accepted the chronological age of 65 years as a definition of “elderly” or older person. For the calculation of the adjusted ROR, the reports were stratified by age as follows: ≤ 59 and ≥ 60 years, because 65 years was categorized into precise 10-year intervals in the JADER database. Neonate, baby, infant, child, young adult, first trimester, second trimester, and third trimester were categorized into the ≤ 59-year-old group. Elderly was categorized into the ≥ 60-year-old group. We excluded the adult and unknown items because these descriptions could not be precisely categorized into the ≤ 59 and ≥ 60-year-old groups. The number of administered drugs was categorized as < 5, 5–9, and ≥ 10 drugs [13,22–25].

### Definition of adverse events

AEs are coded in the "REAC" table in the JADER database according to the terminology preferred by the Medical Dictionary for Regulatory Activities (MedDRA) (www.meddra.org). The standardized MedDRA Queries (SMQ) index is widely accepted and used in analyzing the JADER database. We used the SMQ version 20.0 to extract case reports related to hepatic and renal disorders. We used the following SMQ for *Drug related hepatic disorders—comprehensive search*, *acute renal failure*, *chronic kidney disease*: SMQ codes: 20000006, 20000003, and 20000213, respectively (Table 1). We excluded reports that were incomplete without reporting year, age, or sex information.

**Table 1:** SMQ codes related with hepatic disorder and renal disorder.

	SMQ code	subcategory of SMQ code			Preferred terms (n)	Reported cases (n)
*Hepatic Disorders*	Drug related hepatic disorders—comprehensive search (20000006)	Drug related hepatic disorders—severe events only (20000007)	Hepatitis, non-infectious (20000010)		21	2452
			Liver neoplasms, malignant and unspecified (20000011)	Liver malignant tumours (20000208)	18	493
				Liver tumours of unspecified malignancy (20000209)	2	43
			Liver neoplasms, benign (incl cysts and polyps) (20000012)		9	73
			Hepatic failure, fibrosis and cirrhosis and other liver damage-related conditions (20000013)		92	16347
		Liver related investigations, signs and symptoms (20000008)			96	21349
		Cholestasis and jaundice of hepatic origin (20000009)			17	5882
		Liver-related coagulation and bleeding disturbances (20000015)			40	776
*Renal Disorders*	Acute renal failure (20000003)				50	17622
	Chronic kidney disease (20000213)				172	16081

### Signal detection

The ROR is the authorized pharmacovigilance index [34] and was calculated using two-by-two contingency tables of the presence or absence of a particular drug and a particular AE in the case reports. The ROR is the ratio of the odds of reporting AEs versus all other events associated with the drug of interest compared with the reporting odds for all other drugs present in the database [34]. General qualitative judgments were used for signal detection, which was dependent on the signal indices exceeding a predefined threshold. An association was considered disproportionate when the lower limit of the 95% confidence interval (CI) was > 1 [35]. At least two cases were required to define a signal [36].

The ROR allows for adjustments using multiple-logistic regression analysis and offers the advantage of controlling for covariates [35]. In this analysis, we refined the results with a dedicated correction to detect possible confounders present in the database [35]. We calculated adjusted RORs to control the covariates using a multiple-logistic regression analysis. The reports were stratified by age as follows: ≤ 59 and ≥ 60-year-old group and < 5, 5–9, and ≥ 10 drugs group. To construct the multiple-logistic model, reporting year, stratified age groups, sex, and the number of administered drugs were coded. The following multiple logistic model was used for the analysis:

$$\log(odds)=\beta_{0}+\beta_{1}Y+\beta_{2}S+\beta_{3}A+\beta_{4}N+\beta_{5}S*A+\beta_{6}S*N+\beta_{7}A*N$$

where, Y is the reporting year, A is the stratified age group, S is sex, and N is the number of administered drugs.

The adjusted RORs were calculated using the male, ≤ 59-year-old group, and < 5 drugs as a reference group. We evaluated the effects of explanatory variables using a stepwise method [37] at a significance level of 0.05 (forward and backward, Table 2). A likelihood ratio test was used to evaluate the effects of explanatory variables. A p ≤ 0.05 was considered statistically significant because the difference in the -2log likelihood followed a chi-square distribution with 1 degree of freedom. Chi-Square is the likelihood-ratio chi-square test of the hypothesis that all regression parameters are zero. It is computed by taking twice the difference in negative log-likelihoods between the fitted model and the reduced model that has only intercepts. “Prob > ChiSq” in S4 and S5 Tables is the probability of obtaining a greater chi-square value by chance alone if the specified model fits no better than the model that includes only intercepts. The data analyses were performed using the JMP software version 11.0 (SAS Institute Inc., Cary, NC, USA).

**Table 2:** Multiple-logistic regression analysis.

Adverse events	Variable*	Adjusted ROR^†^ (95% CI)	p-value
Hepatic disorder	Reporting year	0.95 (0.95–0.95)	< 0.0001
	Sex (female)	0.95 (0.93–0.97)	< 0.0001
	Age (≥ 60)	0.86 (0.85–0.88)	< 0.0001
	Number of administered drug (5–9)	1.17 (1.14–1.20)	< 0.0001
	Number of administered drug (≥ 10)	1.14 (1.11–1.18)	< 0.0001
	Sex (female) * Number of administered drug (≥ 10)	1.00 (0.94–1.07)	0.0074
Renal disorder	Reporting year	1.01 (1.01–1.02)	< 0.0001
	Sex (female)	0.81 (0.79–0.83)	< 0.0001
	Age (≥ 60)	1.32 (1.28–1.35)	< 0.0001
	Number of administered drug (5–9)	1.27 (1.24–1.31)	< 0.0001
	Number of administered drug (≥ 10)	1.82 (1.76–1.88)	< 0.0001
	Sex (female) * Age (≥ 60)	1.60 (1.52–1.69)	< 0.0001

* Significant variables selected with stepwise method

^†^ Reporting odds ratio

## Results

**Fig 1:**
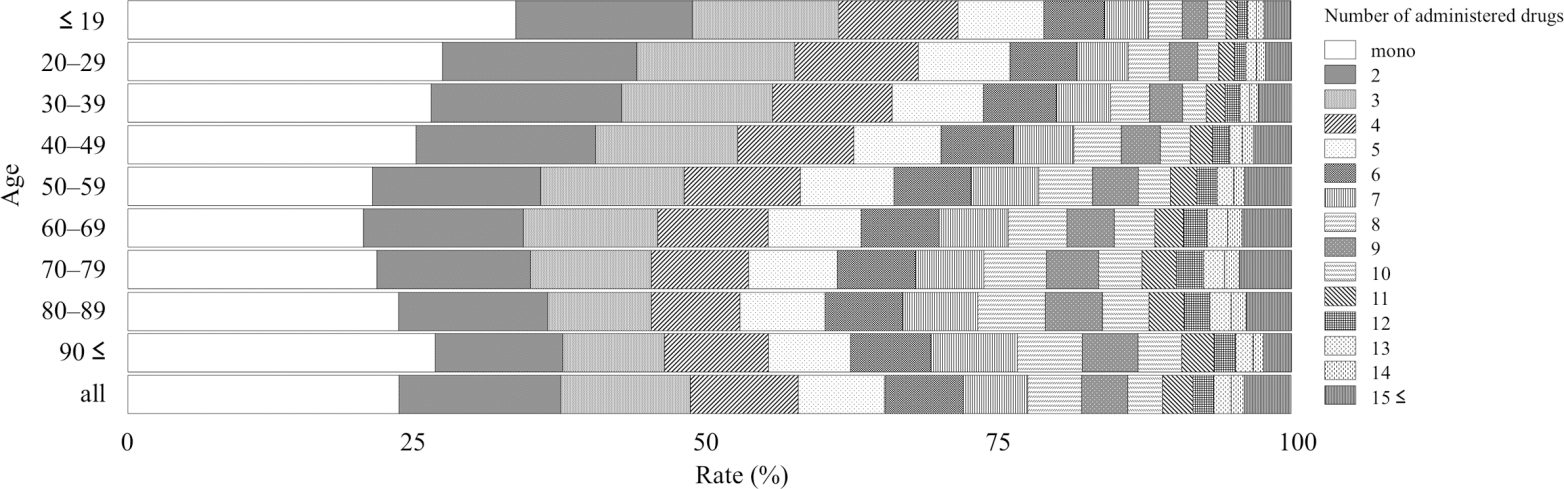
Reporting rate of administered drugs stratified by age for entire dataset.

**Fig 2:**
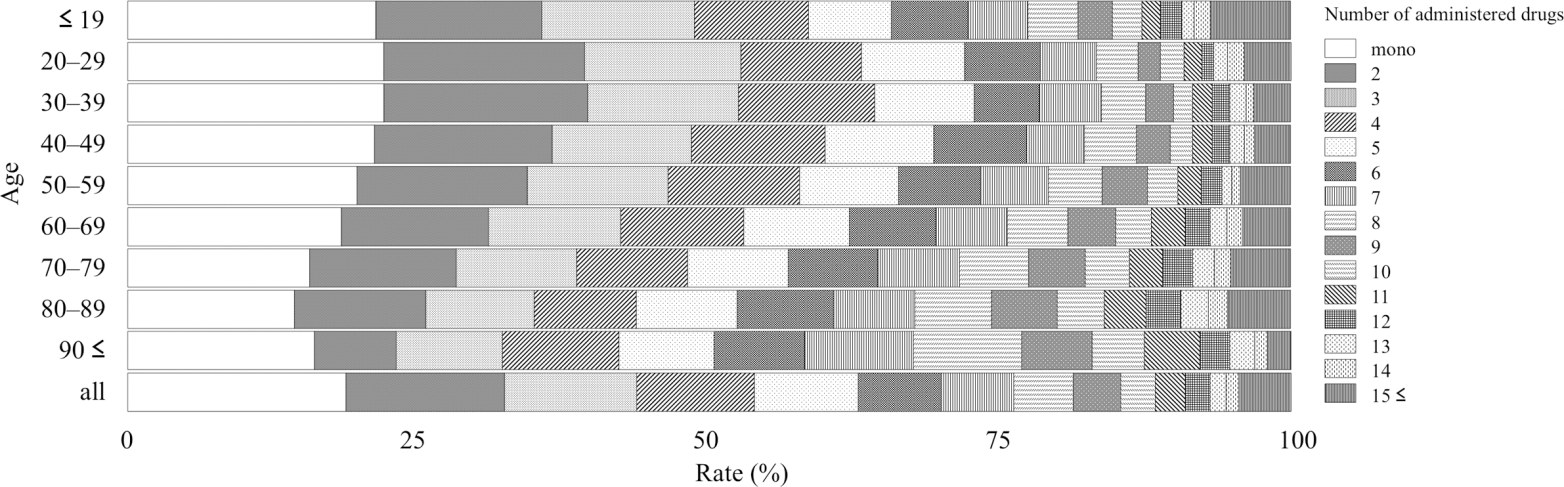
Reporting rate of administered drugs stratified by age for hepatic disorder subset.

**Fig 3:**
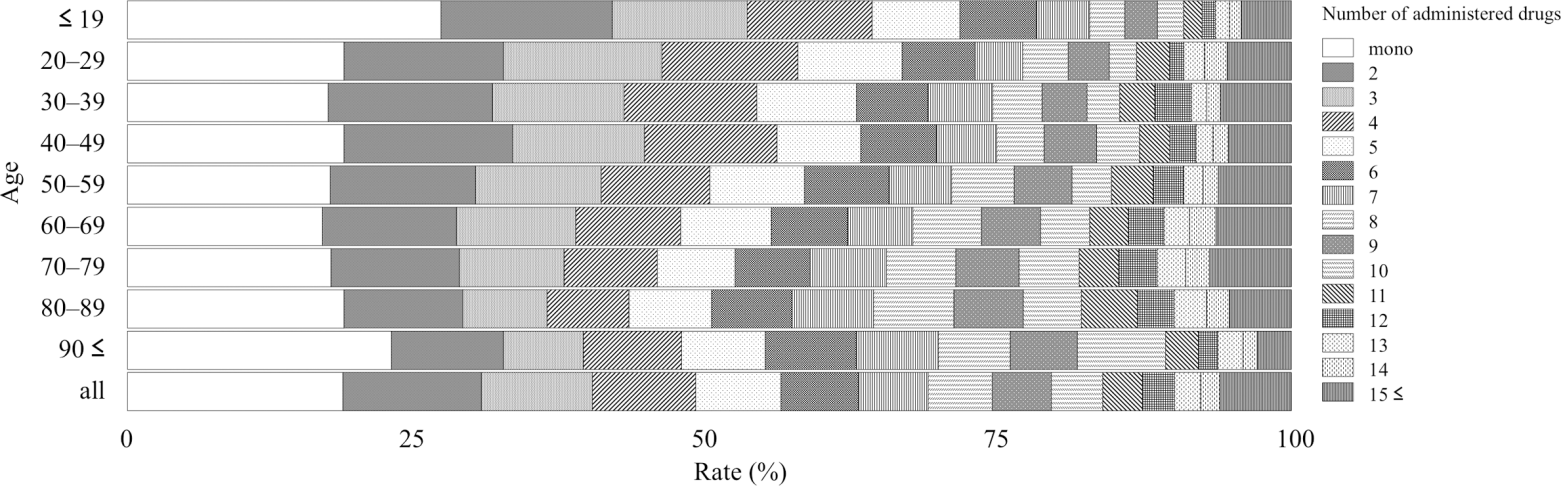
Reporting rate of administered drugs stratified by age for subset data of renal disorder.

The JADER contained 473,487 reports from April 2004 to June 2017, and 42,400 and 29,008 reports were related to hepatic and renal disorders, respectively. The reporting rate stratified by age depended on the number of administered drugs and is summarized as follows: Figs 1–3 show the entire dataset, hepatic disorder, and renal disorder, respectively. The number of reported cases for the 70–79-year-old group was 107,149 (24.8%), which was the highest among all stratified age groups.

For the complete dataset, the reporting rates of AEs in monotherapy with each of the following stratified age groups ≤ 19, 20–29, 30–39, 40–49, 50–59, 60–69, 70–79, 80–89, and ≥ 90 years were 33.5%, 27.2%, 26.2%, 24.9%, 21.2%, 20.5%, 21.5%, 23.4%, and 26.6%, respectively (Fig 1 and S1 Table). The reporting rate of monotherapy was the highest among all stratified age groups in the entire dataset and the subsets for hepatic and renal disorders (Fig 1 and S1 Table, Fig 2 and S2 Table, Fig 3 and S3 Table). In the entire dataset, the monotherapy reporting rates were the highest and lowest in the ≤ 19- and 60–69-year-old groups (33.5 and 20.5%), respectively (Fig 1 and S1 Table).

For the entire dataset, the reporting rate (cases) of the ≥ 5 drugs administered population stratified by age of ≤ 19, 20–29, 30–39, 40–49, 50–59, 60–69, 70–79, 80–89, ≥ 90 years was 28.7% (9,097), 32.2% (5,009), 34.3% (9,092), 37.7% (12,944), 42.1% (24,306), 44.8% (45,054), 46.5% (49,845), 47.3% (24,621), and 44.9% (3,022), respectively. The reporting rate of the population administered more than five drugs, which is commonly referred to as polypharmacy, increased with age and was the lowest and highest in the ≤ 19- and 80–89-year-old groups (28.7 and 47.3%), respectively (Fig 1 and S1 Table).

For the entire dataset, the number of administered drugs was 5.04 ± 4.66 (mean ± standard deviation) and was the highest and lowest in the 70–79- and ≤ 19-year-old groups (5.42 ± 4.79 and 3.93 ± 4.47), respectively. For hepatic disorder, the number of administered drugs was 5.37 ± 4.78 and was the highest and lowest in the 80–89 and 30–39-year-old groups (6.18 ± 4.85 and 4.52 ± 4.20), respectively. For renal disorder, the number of administered drugs was 6.12 ± 5.52 and was the highest and lowest in the 70–79- and ≤ 19-year-old group (6.44 ± 5.35 and 4.90 ± 6.09), respectively. The mean number of administered drugs for renal disorder was higher than that for hepatic disorder.

After excluding incomplete reports that lacked information on the reporting year, age, or sex, 432,337 reports were included in the multiple logistic regression analysis. Using a stepwise logistic regression model, we selected significant variables related to AEs among the reporting year, sex, age, and the number of administered drugs, and examined the interaction between sex, age and the number of administered drugs (Table 2).

For hepatic disorder, the result of the final model indicated that in addition to the significant contribution of reporting year (p < 0.0001), sex (female, p < 0.0001), age (≥ 60 years, p < 0.0001), the number of administered drugs (5–9, p < 0.0001) and the number of administered drugs (≥ 10, p < 0.0001) were observed. The interaction between sex (female) and the number of administered drugs (≥ 10) was also significant (p = 0.0074). The adjusted RORs for the number of administered drugs (5–9, p < 0.0001) and the number of administered drugs (≥ 10) were 1.17 (1.14–1.20) and 1.14 (1.11–1.18), respectively. However, the interaction between age (≥ 60 years) and the number of administered drugs (5–9) or the number of administered drugs (≥ 10) was not significant (data are not shown).

For renal disorder, significant contributions of reporting year (p < 0.0001), sex (female, p < 0.0001), age (≥ 60 years, p < 0.0001), the number of administered drugs (5–9, p < 0.0001), and the number of administered drugs (≥ 10, p < 0.0001) were observed. The interaction between sex (female) and age (≥ 60 years) was also significant (p < 0.0001). The adjusted RORs of age (≥ 60 years), administered drugs (5–9), the number of administered drugs (≥ 10), and interaction between sex (female) and age (≥ 60 years) were 1.32 (1.28–1.35), 1.27 (1.24–1.31), 1.82 (1.76–1.88), and 1.60 (1.59–1.69), respectively. The interaction between age (≥ 60 years) and administered drugs (5–9) or administered drugs (≥ 10) was not significant (data not shown).

We also expressed the results in the heat map of the adjusted RORs obtained from the interaction terms (A*N) from the number of administered drugs and the stratified age groups based on 10-year intervals (Panel E in S1 Fig and S4 Table for hepatic disorder, Panel E in S2 Fig and S5 Table for renal disorder).

## Discussion

The present study suggests that polypharmacy might be more associated with an increased risk of renal disorder than of hepatic disorder based on the adjusted RORs obtained using the multiple-logistic regression technique.

For renal disorder, advanced polypharmacy showed a trend of higher adjusted RORs because the adjusted ROR [1.82 (1.76–1.88)] of administered drugs (≥ 10) group was higher than that [1.27 (1.24–1.31)] of the number of administered drugs (5–9) group (Table 2). Drug interactions play an important role in the effects of polypharmacy. For renal disorder, pharmacokinetic interactions might involve competition for active renal secretion. The frequency of drug interactions depends on the number of administered drugs and the complexity of the regimens [38].

For renal disorder, the lower limit of the 95% CI of adjusted ROR [1.32 (1.28–1.35)] for age (≥ 60 years) was > 1. In the literature, the effect of aging in renal disorders is clear [38]. After the age of 40 years, progressive glomerulosclerosis develops in the kidney, and the number of functioning glomeruli is reduced. The GFR declines by 25–50% between the ages of 20 and 90 years. Generally, women have lower GFRs than men do [39]. According to the adjusted ROR [1.60 (1.52–1.69)] of the interaction between sex (female) and age (≥ 60 years) group, we considered that female patients ≥ 60 years are at a higher risk for renal disorder. However, the lower limit of the 95% CI of adjusted ROR [0.81 (0.79–0.83)] for sex (female) was < 1. Considering this conflicting data, the reason is uncertain.

Objections will no doubt be raised that the interaction between aging and polypharmacy in renal disorder was not statistically significant. However, we constructed the visual heat map of adjusted RORs for AEs from the number of administered drugs and the stratified age group (S2 Fig). For renal disorder, the possibility of an increased risk with concurrent increasing age and increasing number of administered drugs might be a thought-provoking observation in the interpretation of our results.

For hepatic disorder, polypharmacy showed the following slightly higher adjusted RORs: 1.17 (1.14–1.20) of administered drugs (5–9) group, 1.14 (1.11–1.18) of number of administered drugs (≥ 10) group (Table 2). The adjusted RORs of hepatic disorder had lower adjusted RORs than those of renal disorder, which was related to increasing number of administered drugs. We consider that the effect of polypharmacy might be lower in hepatic disorder than it is in renal disorder. In contrast to the result of renal disorder, the lower limit of the 95% CI of adjusted ROR [0.86 (0.85–0.88)] for age (≥ 60 years) was < 1. We do not have a plausible reason. This observation might have occurred because aging does not have a certain effect on metabolism [39]. For cytochrome P450 (CYP) metabolism, the effects of advancing age on the activity of drug-metabolizing enzymes are modest [40]. However, in most studies of human liver samples from adult subjects of varying age, some negative relationships have been observed [40,41]. Glucuronidation and sulfation do not appear to be associated with aging [40]. Multiple factors control hepatic clearance, and the effect of aging differs depending on the controlling factors [39]. No significant interaction was observed between aging and polypharmacy in hepatic disorder (data not shown).

The heat map of adjusted RORs obtained from the interaction terms (A*N) of the number of administered drugs and the stratified age group based on 10-year intervals, revealed a negative correlation between the number of administered drugs and AEs for hepatic disorders (S1 Fig). We do not have a conclusive explanation for this result. In our preliminary analysis, we stratified the reports by the number of administered drug as follows: < 10 drugs (40,470 reports) and ≥ 10 drugs (6,945 reports). In the group of < 10 drugs, the reporting rates of AEs with each of the following SMQ of “drug related hepatic disorders—severe events only (20000007),” “liver-related investigations,” “signs and symptoms (20000008),” “cholestasis and jaundice of hepatic origin (20000009),” and “liver related coagulation and bleeding disturbances (20000015)” were 41.4% (16,744 reports), 44.4% (17,959 reports), 12.8% (5,191 reports), and 1.4% (576 reports), respectively. In the group of ≥ 10 drugs, the reporting rates of AEs with each of the following SMQ of “drug related hepatic disorders—severe events only (20000007),” “liver related investigations,” “signs and symptoms (20000008),” “cholestasis and jaundice of hepatic origin (20000009),” and “liver-related coagulation and bleeding disturbances (20000015)” were 38.4% (2,664 reports), 48.8% (3,390 reports), 9.9% (691 reports), and 2.9% (200 reports), respectively. The result of the chi-square test showed there was a significant difference in the reporting number profiles stratified with each SMQ between the < 10 drugs administered group and ≥ 10 drugs administered group (p < 0.0001). We could not find any persuasive explanation for our result at this time. In evaluating SRS, unadjusted confounding factors should be considered. The covariates should be evaluated with respect to a variety of patient backgrounds using well-organized epidemiologic studies in the future.

Media attention and publicity resulting from advertising or regulatory actions such as safety information from the PMDA might influence the JADER database reporting based on the year of reporting [42,43]. In this study, we adjusted the ROR for the variable of the reporting year. Although the likelihood ratio test was significant (p < 0.0001), the value of the adjusted ROR for reporting year was nearly one. We considered that the effects of reporting year were small.

Polypharmacy is related to increased risk of being administered potentially inappropriate medications (PIM) [44,45]. Both polypharmacy and PIMs increase AEs, worsen physical functions, and cause excessive use of health facilities [46,47]. PIMs were first devised and publicized by Beers et al. [48] for nursing home residents, were subsequently expanded to include older adults in all settings in 1991, and last updated in 2015 [49]. Other PIM criteria including screening tool of older people’s prescriptions (STOPP)/screening tool to alert doctors to right treatment (START) have been used [50]. These criteria are suitable for determining the appropriateness of prescriptions. The Beers criteria provide a list of drugs that the panel of experts considered particularly problematic for older patients [1]. The STOPP and START tools identify inappropriate medications in elderly, including drug-drug and drug-disease interactions, drugs that increase the risk of falls, and drugs that duplicate therapy [50]. The Beers and STOPP tools are used to address over- and misuse of medications. The START tool allows the detection of potentially inappropriate drug omissions [51]. However, information on the AE risk dependence on the number of administered drugs is insufficient in these criteria [52]. Few studies have examined the relationship between the number of administered drugs and AE in the context of age-group stratification. Our results provide valuable insights into prescribing drugs to older patients using real-world clinical setting.

The analysis using SRS such as the JADER database has several limitations that are worth noting. The SRS is subject to over-reporting, under-reporting, missing data, exclusion of healthy individuals, lack of a denominator, and the presence of confounding factors [34]. In general, crude RORs are inappropriate for inferring the relative strength of association of factors and only offer an approximate indication of signal strength [34]. On the other hand, the ROR is an applicable technique that allows the control of covariates using a multiple-logistic regression analysis and can be used for the detailed analysis of interaction terms [30,53–58]. Multiple-logistic regression analysis mitigates the effect of confounding factors, thereby enhancing the robustness of the result [53,54]. Despite the limitations inherent to SRSs, we obtained reasonable results that complement or corroborate those reported in the literature [2,17].

An observation could be made that the polypharmacy- and aging-related risks are different for each AE and drug. However, data subsetting using the SMQs such as *hepatic* or *renal disorders* might be useful for evaluating aging-, polypharmacy-, or aging-polypharmacy-related AE associations in the interpretation of results, because the subsets consist of a population of patients that share a set of commonly administered drugs, risk factors, or diseases. This approach might be applicable for the simultaneous AE risk evaluation of polypharmacy and aging. We considered that the subset analyses focusing on drugs of interest, such as antibiotics that are expected to cause renal disorder, might be valuable. We expect our study to make a valuable contribution to the information available for clinicians and would facilitate the improvement of polypharmacy management.

## Conclusion

To the best of our knowledge, this study was the first to evaluate the AE profiles related to aging and polypharmacy using the JADER database. We demonstrated that polypharmacy might be more closely associated with an increased risk of *renal disorder* than *hepatic disorder* because the adjusted RORs in *renal disorder* were higher for those who were on polypharmacy than for those who were not. Age-related increase in renal disorder is well-known. Thus, older patients on polypharmacy treatments should be closely monitored for renal disorder. Our results are useful for improving the management of polypharmacy. Further research is needed to determine the specific associations between polypharmacy and AEs for each drug.

## Supporting information

S1 Table Reporting rate of administered drugs stratified by age for entire dataset. (XLSX)

S2 Table Reporting rate of administered drugs stratified by age for subset data of hepatic disorder. (XLSX)

S3 Table Reporting rate of administered drugs stratified by age for subset data of renal disorder. (XLSX)

S4 Table Adjusted reporting odds ratio (ROR) for adverse events (AEs) of hepatic disorder. log(*odds*) = β_0_ + β_1_Y + β_2_S + β_3_A + β_4_N + β_5_S * A + β_6_S * N + β_7_A * N. Reports were stratified by age as follows: ≤ 19, 20–29, 30–39, 40–49, 50–59, 60–69, 70–79, 80–89, and ≥ 90 years. Adjusted RORs were calculated using male, 19-year-old ≤ group, and one drug as a reference group.
(XLSX)

S5 Table Adjusted reporting odds ratio (ROR) for adverse events (AEs) of renal disorder. log(*odds*) = β_0_ + β_1_Y + β_2_S + β_3_A + β_4_N + β_5_S * A + β_6_S * N + β_7_A * N. Reports were stratified by age as follows: ≤ 19, 20–29, 30–39, 40–49, 50–59, 60–69, 70–79, 80–89, and ≥ 90 years. Adjusted RORs were calculated using male, 19-year-old ≤ group, and one drug as a reference group.
(XLSX)

S1 Fig Heat map of hepatic disorder. A) Number of the administered drugs, B) Age, C) Number of administered drugs * Sex (female), D) Age * Sex (female), E) Number of administered drugs * Age. log(*odds*) = β_0_ + β_1_Y + β_2_S + β_3_A + β_4_N + β_5_S * A + β_6_S * N + β_7_A * N. Reports were stratified by age as follows: ≤ 19, 20–29, 30–39, 40–49, 50–59, 60–69, 70–79, 80–89, and ≥ 90 years. Adjusted RORs were calculated using male, 19-year-old ≤ group, and one drug as a reference group.
(TIF)

S2 Fig Heat map of renal disorder. A) Number of the administered drugs, B) Age, C) Number of administered drugs * Sex (female), D) Age * Sex (female), E) Number of administered drugs * Age. log(*odds*) = β_0_ + β_1_Y + β_2_S + β_3_A + β_4_N + β_5_S * A + β_6_S * N + β_7_A * N. Reports were stratified by age as follows: ≤ 19, 20–29, 30–39, 40–49, 50–59, 60–69, 70–79, 80–89, and ≥ 90 years. Adjusted RORs were calculated using male, 19-year-old ≤ group, and one drug as a reference group.
(TIF)
