# Supplementary figures and images for: Analysis of polypharmacy effects in older patients using Japanese Adverse Drug Event Report database
Source: PLoS One. 2017 Dec 21;12(12):e0190102. doi: 10.1371/journal.pone.0190102 (PMC5739473; doi:10.1371/journal.pone.0190102)

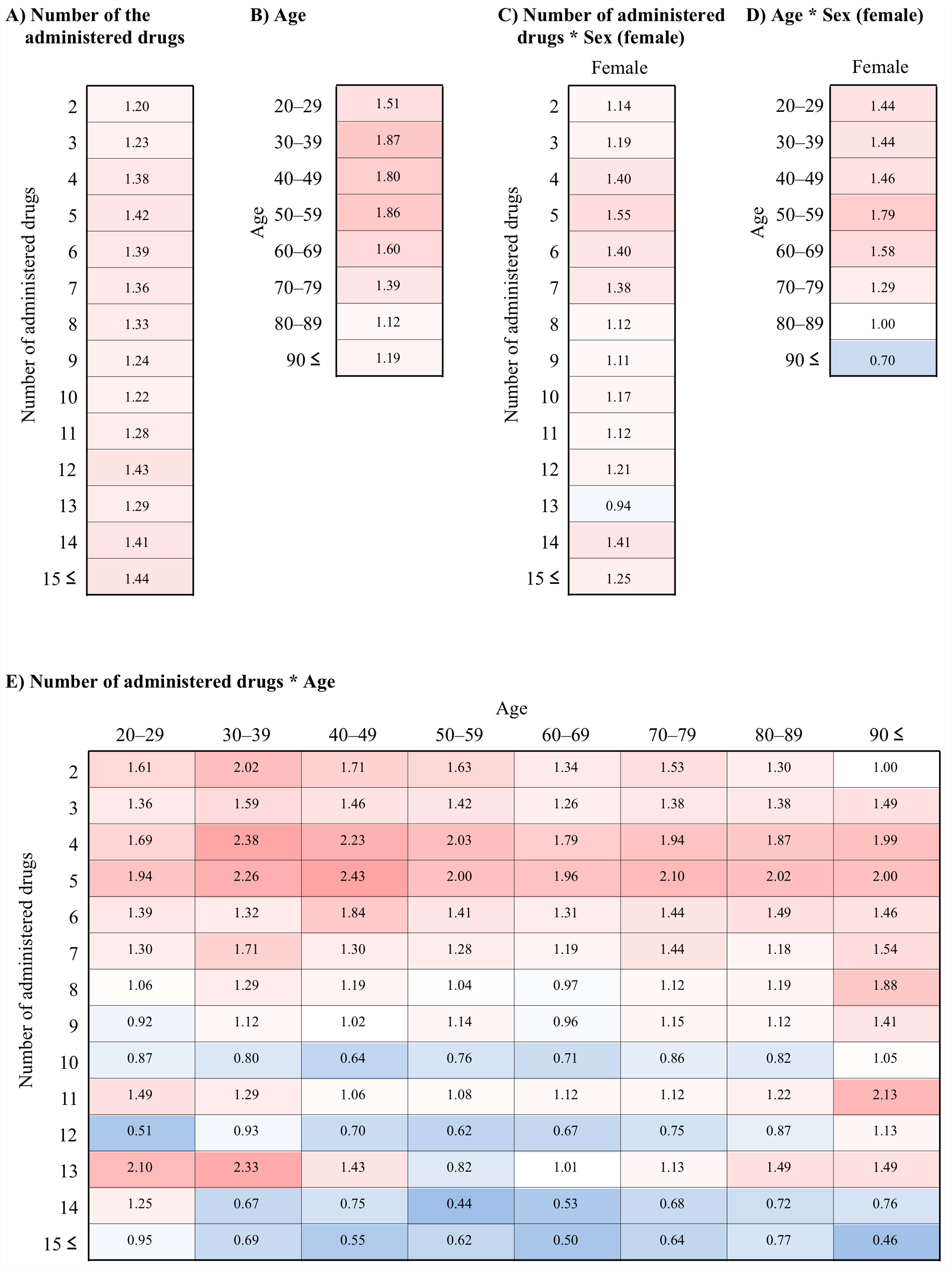

Supplement: S1 Fig — A) Number of the administered drugs, B) Age, C) Number of administered drugs * Sex (female), D) Age * Sex (female), E) Number of administered drugs * Age. log(odds) = β0 + β1Y + β2S + β3A + β4N + β5S * A + β6S * N + β7A * N. Reports were stratified by age as follows: ≤ 19, 20–29, 30–39, 40–49, 50–59, 60–69, 70–79, 80–89, and ≥ 90 years. Adjusted RORs were calculated using male, 19-year-old ≤ group, and one drug as a reference group. (TIF) [file pone.0190102.s006.tif]

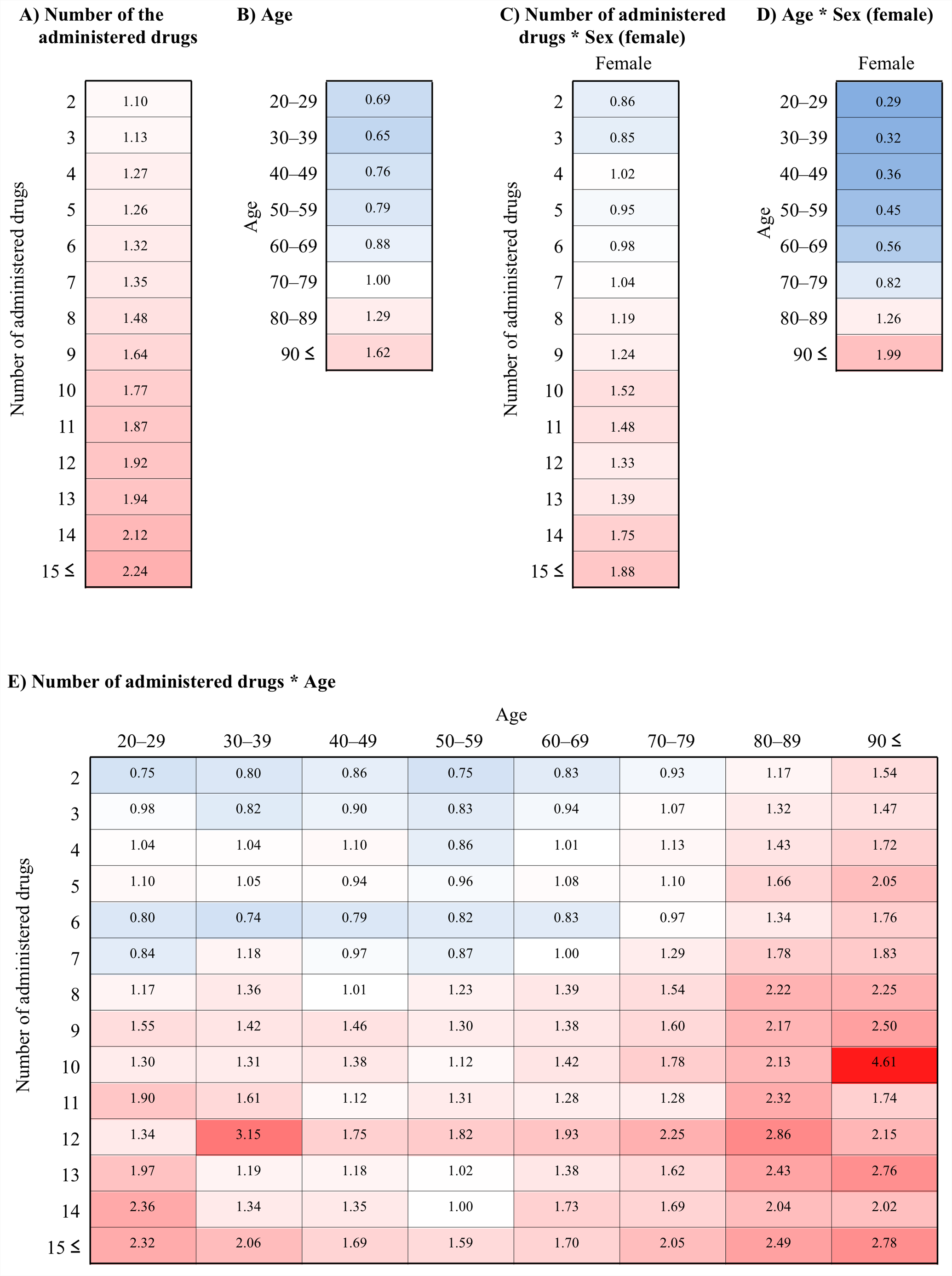

Supplement: S2 Fig — A) Number of the administered drugs, B) Age, C) Number of administered drugs * Sex (female), D) Age * Sex (female), E) Number of administered drugs * Age. log(odds) = β0 + β1Y + β2S + β3A + β4N + β5S * A + β6S * N + β7A * N. Reports were stratified by age as follows: ≤ 19, 20–29, 30–39, 40–49, 50–59, 60–69, 70–79, 80–89, and ≥ 90 years. Adjusted RORs were calculated using male, 19-year-old ≤ group, and one drug as a reference group. (TIF) [file pone.0190102.s007.tif]
